# Suitability of ultrasound-guided fine-needle aspiration biopsy for transcriptome sequencing of the canine prostate

**DOI:** 10.1038/s41598-019-49271-1

**Published:** 2019-09-13

**Authors:** H. Thiemeyer, L. Taher, J. T. Schille, L. Harder, S. O. Hungerbuehler, R. Mischke, M. Hewicker-Trautwein, Z. Kiełbowicz, B. Brenig, E. Schütz, J. Beck, H. Murua Escobar, I. Nolte

**Affiliations:** 1 Small Animal Clinic, University of Veterinary Medicine Hannover, Foundation, Hannover, Germany; 2 Department of Haematology/Oncology/Palliative Care, Rostock University Medical Centre, Rostock, Germany; 3 Division of Bioinformatics, Department of Biology, Friedrich-Alexander-Universität Erlangen-Nürnberg, Erlangen, Germany; 4 Institute of Pathology, University of Veterinary Medicine Hannover, Foundation, Hannover, Germany; 5 Department and Clinic of Veterinary Surgery, Faculty of Veterinary Medicine, Wroclaw University of Environmental and Life Sciences, Wrocław, Poland; 6 University of Göttingen, Institute of Veterinary Medicine, Göttingen, Germany; 7 Chronix Biomedical, Göttingen, Germany

**Keywords:** Transcriptomics, Prostate cancer, Prostate

## Abstract

Ultrasound-guided fine-needle aspiration (US-FNA) biopsy is a widely used minimally invasive sampling procedure for cytological diagnosis. This study investigates the feasibility of using US-FNA samples for both cytological diagnosis and whole transcriptome RNA-sequencing analysis (RNA-Seq), with the ultimate aim of improving canine prostate cancer management. The feasibility of the US-FNA procedure was evaluated *intra vitam* on 43 dogs. Additionally, aspirates from 31 euthanised dogs were collected for standardising the procedure. Each aspirate was separated into two subsamples: for cytology and RNA extraction. Additional prostate tissue samples served as control for RNA quantity and quality evaluation, and differential expression analysis. The US-FNA sampling procedure was feasible in 95% of dogs. RNA isolation of US-FNA samples was successfully performed using phenol-chloroform extraction. The extracted RNA of 56% of a subset of US-FNA samples met the quality requirements for RNA-Seq. Expression analysis revealed that only 153 genes were exclusively differentially expressed between non-malignant US-FNAs and tissues. Moreover, only 36 differentially expressed genes were associated with the US-FNA sampling technique and unrelated to the diagnosis. Furthermore, the gene expression profiles clearly distinguished between non-malignant and malignant samples. This proves US-FNA to be useful for molecular profiling.

## Introduction

Numerous diagnostic approaches for canine prostate diseases have been developed in the last few decades, substantially improving the detection of benign conditions^1–3^. However, there is still a lack of information on the molecular mechanisms underlying canine prostate diseases, which is especially relevant to the early diagnosis and management of canine prostatic tumours^4^. Dogs with prostate cancer (PCa) are often diagnosed at an advanced stage, which is associated with poor prognosis and limited therapeutic options^4,5^. Although the reported prevalence of prostate malignancies is low (0.2–0.6%)^6,7^, the incidence is likely to be underestimated due to the absence of reliable biomarkers and the lack of biopsies on asymptomatic dogs^4^. In addition, aside from humans, dogs are the only non-human species to naturally develop PCa sharing many clinical properties^4,8^ and will often have metastases^9,10^, similar to men^8,11^. This highlights the importance of characterising the transcriptomic landscape of canine PCa. Next-generation sequencing (NGS) of RNA (RNA-Seq) has enabled comprehensive insights into cancer-relevant mechanisms^12,13^ and is a well-established method for differentiating between neoplastic diseases^14^. Compared to conventional “gene by gene” approaches, RNA-Seq has several advantages: it is fast, cost-efficient and provides simultaneous information on several thousands of genes^13^. There is little doubt that RNA-Seq has the potential of becoming a routine diagnostic tool – at least in human medicine^15^. And even if the cost and complexity of the analysis may prevent this in veterinary practice in the immediate future^16,17^, RNA-Seq data already serve as a solid basis for fostering new diagnostic and therapeutic approaches^13,16^. Indeed, RNA-Seq has already been utilised in dogs to address clinical issues in different organs^18–22^. And while no genome-wide transcriptome analysis have been conducted for canine PCa, there is a growing body of information on the transcriptomic landscape of human PCa^23–26^. NGS allows the analysis of the transcriptome from minimal amounts of sampling material^27^. This is of special interest in clinical routine diagnostics, where representative sample material from tumours or organs must be obtained *intra vitam*. Meeting the requirements of a minimal invasive sampling procedure^2^, aspirates collected by ultrasound-guided fine-needle aspiration (US-FNA) biopsy are regularly employed in the cytological diagnosis of canine prostate diseases^28^ and other diagnostic approaches, such as flow cytometric phenotyping^29^. In this context, ultrasonographic parameters such as prostate size and differences in echogenicity within the parenchyma are used to identify lesions^3^ and guide the fine-needle to aspirate cells from representative areas^30^. The cells targeted and collected by the US-FNA procedure normally undergo cytological diagnosis^28^, but are also viable for molecular profiling^31,32^.

The aim of the present study was to determine the suitability of aspirates derived from canine prostate US-FNA for molecular diagnostic. In pursuing this aim, the following questions were investigated: (a) the technical feasibility of the canine prostate US-FNA procedure as a sampling tool for cytological diagnosis and laboratory workflow; (b) the quantity and quality of the RNA isolated from US-FNA subsamples; (c) the impact of methodological contamination (e.g., with blood) in US-FNA aspirates on the transcriptome analysis; and (d) the similarities and differences in the molecular profiles of malignant and non-malignant US-FNA samples compared to those of prostate tissue samples.

## Results

### Feasibility of fine-needle aspiration

**Figure 1:**
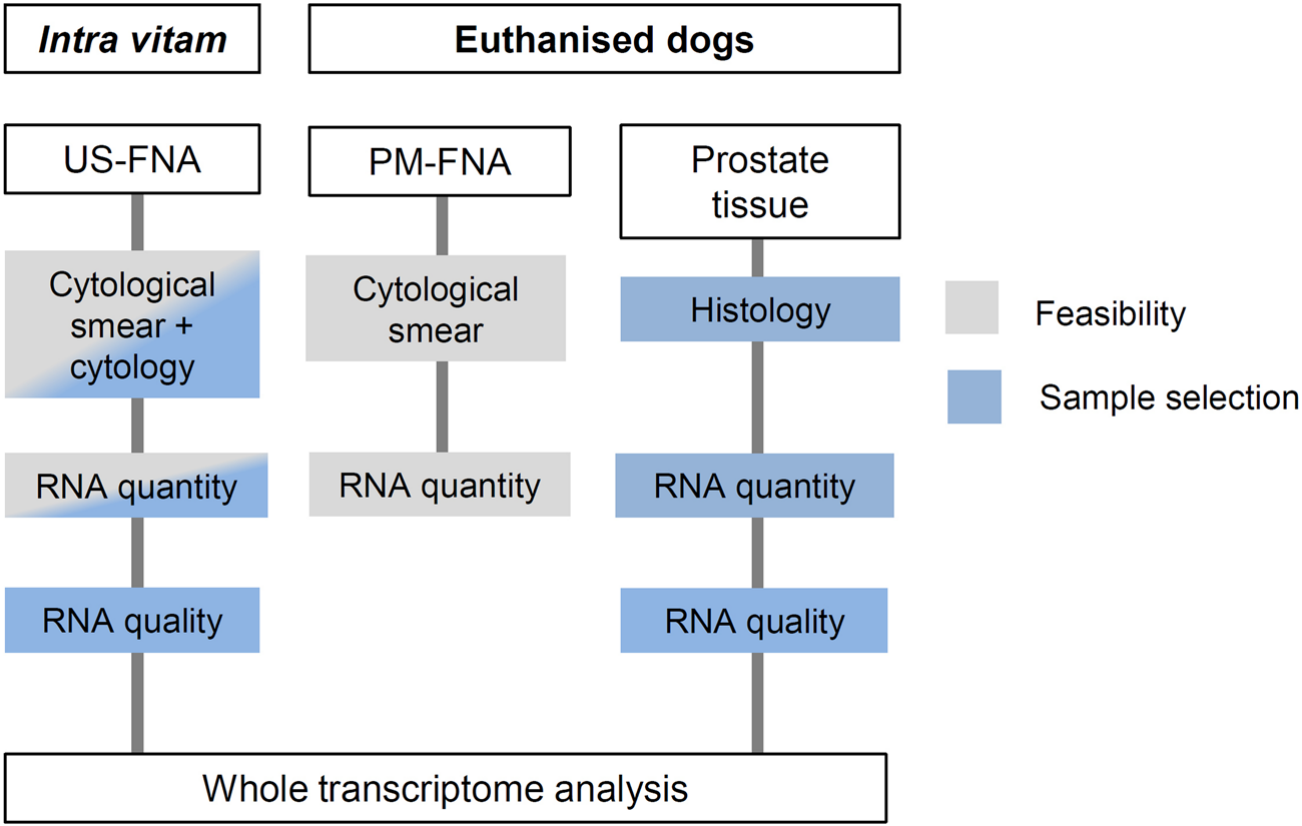
Study design. Flow chart of study workflow and sample selection. Ultrasound-guided fine-needle aspiration (US-FNA) and *post mortem* fine-needle aspiration (PM-FNA) samples used for evaluation of the feasibility of the procedure are highlighted in grey. On the basis of diagnosis, RNA quantity and quality, a subset of US-FNA and prostate tissue samples was used for RNA-Seq (blue).

First, the *post mortem* FNA (PM-FNA) training set (see Fig. 1 and Materials and Methods) was used to establish the sampling strategy. The procedure was simple to perform, except for restricted needle movement within the small and solid prostate of six castrated dogs with no evidence of prostate disease. Next, the feasibility of the sampling strategy was evaluated *intra vitam*, using ultrasound imaging to guide the needle to the area of concern (US-FNA, see Fig. 1). The 43 dogs subjected to the US-FNA procedure (see Table 1 and Materials and Methods) had a median age of 7.8 years and a mean body weight of 29.3 kg (range 6.5 to 75.0 kg). Their ultrasonographic examination identified 33 as non-malignant and ten as suspicious for canine PCa. Forty-one dogs (95%) underwent the US-FNA procedure without sedation; the US-FNA procedure was unable to be carried out for one American Pit Bull Terrier due to defensive movement and one Alsatian dog due to anxious behaviour (see Table 1). Sedation was not performed, the US-FNA sampling procedure was discontinued, and these two dogs were excluded from further analysis. Out of the 41 dogs, four (10%) showed complications after undergoing the US-FNA procedure. Two dogs showed transient haematuria for one day and one dog had symptoms of stranguria for two days. One dog with massive hyperplastic changes of the prostate showed moderate bleeding after the US-FNA procedure; resulting haematoma was no longer detectable in the follow-up ultrasound 16 days later.

**Table 1:** Study Overview.

		*Intra vitam*		*Post mortem*		Total
		male intact	neutered	male intact	neutered	
Sample details	Dogs	37	6	27	14	**84**
	US-FNA	35	6 + 1**			41 + 1**
	PM-FNA	1*	1*	20	9	**31**
	Prostate tissue	1*	1*	27	14	**43**
	No sample	2				**2**
		**Cytology**		**Histopathology**		
Diagnosis	Normal/ hyperplastic	28		20		**48**
	Prostate carcinoma	1	3	3	6	**13**
	Suspicious as carcinoma		1			**1**
	Metastasis				2	**2**
	Inflammation	3		3	1	**7**
	Atrophy			1	5	**6**
	Non-diagnostic sample	3	2			**5**
RNA quantity	US-FNA	42				**42**
	PM-FNA	2		29		**31**
	Tissue	2		41		**43**
RNA quality	Non-malignant	12		9		**21**
	Malignant	4		9		**13**
RNA-Seq	Non-malignant	5		9		**14**
	Malignant	2		9		**11**

Sample details, including the number of male dogs used for *post mortem* fine-needle aspiration (PM-FNA) and *intra vitam* ultrasound-guided fine-needle aspiration (US-FNA) procedure as well as prostate tissue samples. Additionally, number of dogs with histopathological or cytological diagnosis, number of samples used to determine the RNA quantity, RNA quality and RNA-sequencing (RNA-Seq).

*Dogs euthanised due to prostate carcinoma; additional PM-FNA and matching prostate tissue sample.

**Additional US-FNA sample from one euthanised dog.

Aspirates were divided into two subsamples: one for preparing cytological smears and one isolating RNA (see Fig. 1). For 29 out of the 41 dogs that underwent the US-FNA procedure (71%), a single aspirate was sufficient for both preparing a diagnostic cytological smear and isolating RNA. For the remaining 12 dogs (29%), the US-FNA procedure had to be repeated to provide adequate amounts of material (see Table 2).

**Table 2:** Samples collected by ultrasound-guided fine-needle aspiration (US-FNA).

Dog ID	Sample ID	Reproductive status	Number of US-FNA procedures	Cellularity of smears	Diagnosis	Blood Scoring	RNA (ng/µl)	RIN	RNA-Seq
1*	US-1	m	1	S	PCa	—	N/A	—	—
2**	US-2	n	3	S	PCa	—	N/A	—	—
	US-3					4	59	N/A	y
3	US-4	n	1	S	PCa	2	44.1	*6.9*	y
4	US-5	m	1	S	nm	0	2.3	—	—
5	US-6	n	2	S	PCa	1	9.1	N/A	—
6	US-7	m	2	S	nm	3	35.6	6.2	y
7	US-8	m	3	S	nm	4	37.5	—	—
8	US-9	m	2	S	nm	4	91	—	—
9	US-10	n	2	S	PCa	3	21.7	1.9	—
10	US-11	m	1	S	nm	3	33.2	—	—
11	US-12	m	1	S	nm	4	85	—	—
12	US-13	m	1	S	nm	2	90	6	y
13	US-14	m	1	S	nm	3	54	—	—
14	US-15	m	3	S	nm	4	61	6.1	y
15	US-16	m	1	S	nm	3	74	5.7	—
16	US-17	m	1	Nd	N/A	3	69	—	—
17	US-18	m	1	S	nm	2	66	—	—
18	US-19	m	2	S	nm	3	20.9	—	—
19	US-20	m	2	S	nm	3	41.1	—	—
20	US-21	m	1	S	nm	2	11.2	1	—
21	US-22	m	2	S	nm	2	8.2	—	—
22	US-23	m	1	Nd	N/A	3	18.7	—	—
23	US-24	m	1	S	nm	0	26.6	—	—
24	US-25	m	1	S	nm	3	54	—	—
25	US-26	m	1	S	nm	2	30.1	4.2	—
26	US-27	m	1	S	nm	3	56	—	—
27	US-28	m	1	S	nm	4	47.8	N/A	—
28	US-29	m	1	S	nm	2	30	—	—
29	US-30	m	1	S	nm	3	72	—	—
30	US-31	m	1	Nd	N/A	0	N/A	—	—
31	US-32	m	1	S	nm	3	60	6.7	y
32	US-33	n	1	Nd	N/A	2	11.3	1.5	—
33	US-34	m	1	S	nm	2	51	—	—
34	US-35	m	1	S	nm	2	68	—	—
35	US-36	m	1	S	nm	3	378.6	—	—
36	US-37	m	1	S	nm	3	48	—	—
37	US-38	m	1	S	nm	3	24	3.2	—
38	US-39	m	1	S	nm	3	99	5.7	—
39	US-40	m	2	S	nm	4	601.1	7.2	y
40	US-41	n	1	Nd	N/A	3	98	—	—
41	US-42	m	3	S	nm	3	72	—	—
42***	N/A	m	—	—	—	—	—	—	—
43***	N/A	m	—	—	—	—	—	—	—

Sample details, including: identification number (ID) of the dogs and the corresponding sample ID; reproductive status, male (m) and neutered (n); cytological smears, sufficient (S) and non-diagnostic (Nd); diagnosis, prostate carcinoma (PCa) and non-malignant (nm); semi-quantitative scoring of blood contamination as follows: 0 = no, 1 = minimal, 2 = weak, 3 = moderate, 4 = strong blood contamination; RNA quantity; RNA integrity number (RIN); and samples used for RNA-Seq.

*Dog euthanised due to prostate carcinoma enabled additional sampling opportunity.

**Dog euthanised due to prostate carcinoma enabled additional sampling including one additional ultrasound-guided fine-needle aspiration.

***US-FNA procedure was omitted.

N/A, not available.

y, US-FNA samples selected for RNA-Seq.

### Cytology and histopathology

The cellularity of the cytological smears collected *intra vitam* by the US-FNA procedure was sufficient for the cytological diagnosis of 36 of the 41 dogs (88%, see Table 2). The cytological smears were diagnosed as normal to hyperplastic (n = 28, 68%), inflammation (n = 3, 7%) or malignant (n = 4; 10%) (Table 1). One cytological smear showed sporadic criteria of malignancy and was consequently categorised as suspicious for prostate carcinoma. For the remaining five dogs (two neutered and three intact dogs), the cytological smears were non-diagnostic. The quality of the cytological smears was lowered by the presence of necrotic cells in one sample and blood contamination in ten samples (24%). The cytological diagnosis of two dogs was confirmed by histopathology after euthanasia due to an advanced stage of prostate carcinoma (see Table 1).

In addition to the samples collected by the US-FNA procedure, a set of prostate tissue samples was collected from 41 euthanised dogs (see Materials and Methods) and histopathologically diagnosed as normal to hyperplastic (n = 20, 49%), inflammation (n = 4, 10%), prostatic atrophy (n = 6, 15%), prostate carcinoma (n = 9, 22%) or metastasis (n = 2, 5%) from a transitional cell carcinoma of the urinary bladder or lymphoma (see Table 1).

### RNA quantity

RNA quantity was evaluated on 42 US-FNA samples from 41 dogs, 31 PM-FNA aspirates from 31 dogs, and 43 tissue samples from 43 dogs (see Table 1).

**Figure 2:**
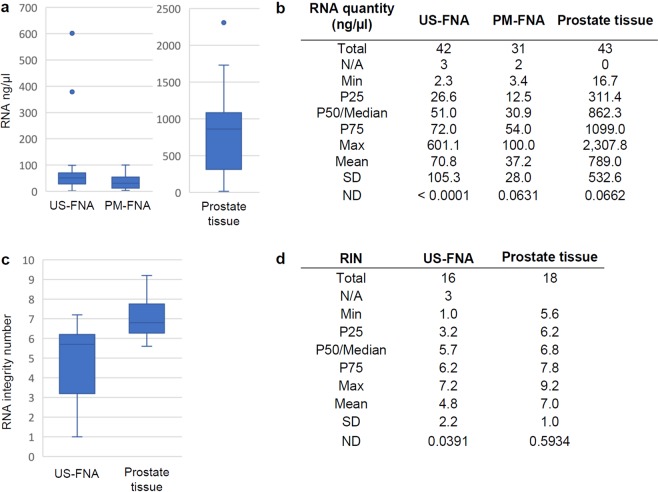
RNA quantity and quality in different sample types. Details on RNA quantity are shown in (**a**,**b**) including the standard deviation (SD). Data on RNA integrity number (RIN) are present in (**c**,**d**) including the SD. The normal distribution (ND) was calculated by the Shapiro-Wilk test. The boxes (**a**,**c**) enclose samples within the 25th to the 75th percentiles. The lowest and largest values are visualised using bars. The horizontal line represents the median.

For two US-FNA samples, RNA isolation was attempted using column-based extraction (see Materials and Methods) but failed due to the presence of clotted blood. Semi-quantitative macroscopic evaluation of the remaining 40 US-FNA samples (see Materials and Methods) revealed that 65% showed moderate to strong blood contamination, 28% had weak to nearly invisible blood additions and 8% exhibited no signs of blood contamination. For this reason, RNA isolation from these samples was performed using the phenol-chloroform extraction method, which performed well in all but one US-FNA sample, resulting in a mean of 70.8 ng/µl of total RNA (range: 2.3 ng/µl to 601.1 ng/µl, see Table 2 and Fig. 2). For comparison, RNA quantification on the PM-FNA training set yielded values of the same order of magnitude. Specifically, total RNA was quantifiable in 29 out of 31 PM-FNA samples, with a mean of 37.2 ng/µl of total RNA (range: 3.4 ng/µl to 100.0 ng/µl, see Fig. 2), which was not significantly different from that observed for US-FNA samples.

**Figure 3:**
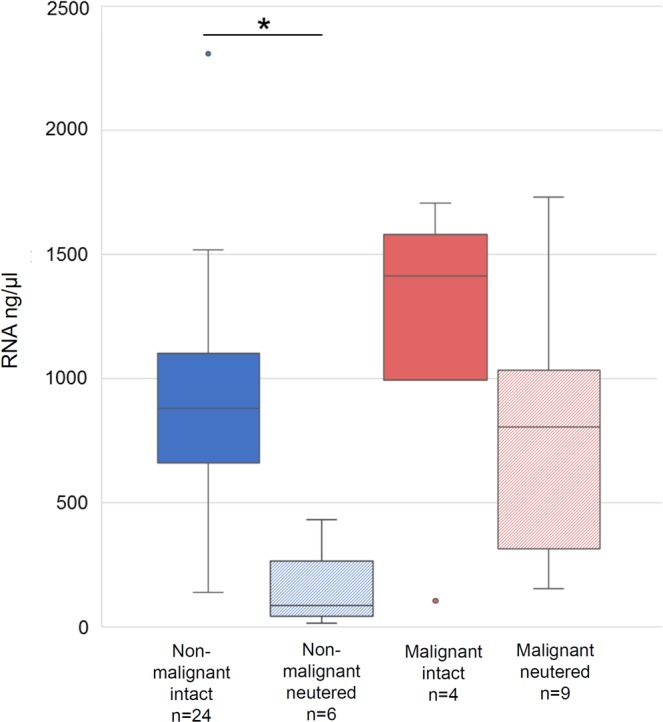
RNA quantity in non-malignant and malignant prostate tissue samples from intact and neutered male dogs. The asterisk indicates a significant difference (p < 0.05) between male intact and neutered dogs in non-malignant prostate tissue samples. The boxes enclose the 25th to the 75th percentiles. The lowest and largest values are visualised using bars. The horizontal line represents the median.

As expected given the amount of starting material involved (10–20 mg, see Materials and Methods), RNA isolation from tissue resulted in a mean of 789.0 ng/µl of total RNA (range: 16.7 to 2,307.8 ng/µl), which was significantly higher (p < 0.05) than those obtained for the US-FNA samples and PM-FNA training set (see Fig. 2). Furthermore, the neutering state of the dogs had an effect on the RNA quantity of non-malignant tissues (see Fig. 3). Indeed, non-malignant tissue from neutered dogs had a significantly lower (p < 0.05) mean compared to that from intact dogs (means 162.1 ng/µl and 897.5 ng/µl, respectively). No significant differences were observed between malignant tissue from neutered dogs and malignant tissue from intact dogs.

In total, 93% of US-FNA, 94% of PM-FNA and 100% of tissue samples met the quantity requirements for RNA-Seq (see Materials and Methods).

### RNA quality

Based on clinical data, histopathological and cytological diagnosis (see Fig. 1), 16 US-FNA (12 non-malignant and four malignant) and 18 tissue (nine non-malignant and nine malignant) samples were selected for evaluation of RNA quality.

The RNA integrity numbers (RIN) were more consistent for tissue samples (5.6 to 9.2, see Fig. 2) than for US-FNA samples (1.0 to 7.2). In total, nine (56%) US-FNA samples met the quality requirements for RNA-Seq (RIN ≥ 5.5, see Materials and Methods and Table 2). Of these, one malignant sample had no detectable RIN value, but an acceptable electropherogram and was therefore used for further analysis. Among samples discarded from further analysis, five (three non-malignant and two malignant) showed low RNA quantities (<25 ng/µl). For comparison, RIN values for tissue samples ranged from 5.6 to 7.6 (mean 6.6) for non-malignant samples and from 5.6 to 9.2 (mean 7.4) for malignant samples without significant difference.

### Comparative molecular profiling of US-FNA and tissue samples

**Figure 4:**
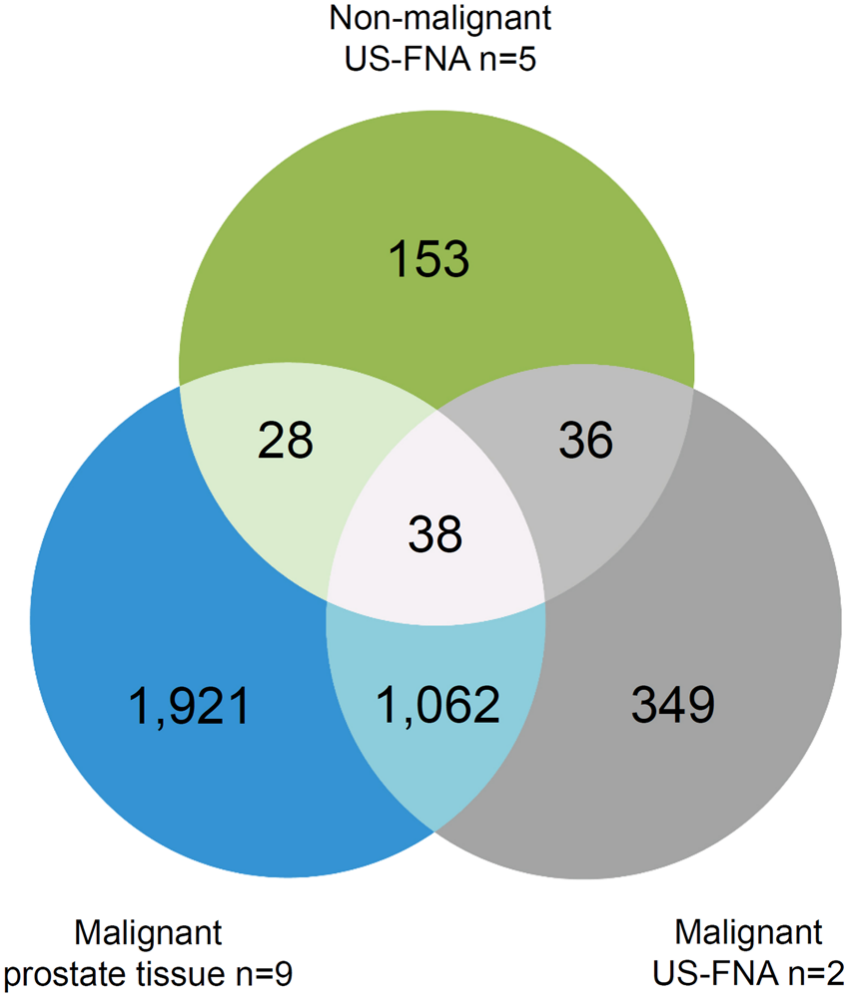
Venn diagram of differentially expressed genes (DEGs) in different sample types relative to control. The venn diagram visualises similarities and differences of DEGs in non-malignant ultrasound-guided fine-needle aspiration (US-FNA) (green), malignant US-FNA (grey) and malignant prostate tissue (blue) sample groups compared with non-malignant prostate tissue samples control. A total of 3,587 genes were consistently differentially expressed in non-malignant US-FNA, malignant prostate tissue or malignant US-FNA samples.

Final sample selection was performed with focus on clinical diagnosis, RNA quantity and quality. A total of seven US-FNA and 18 tissue samples were used for RNA-Seq library preparation (see Fig. 1 and Table 1). Bioinformatics analysis of RNA-Seq data revealed 3,587 differentially expressed genes (DEGs) when comparing non-malignant aspirates of US-FNA (n = 5), malignant tissue (n = 9) and malignant US-FNA aspirates (n = 2) to the non-malignant tissue control group (n = 9, see Fig. 4 and Materials and Methods).

**Figure 5:**
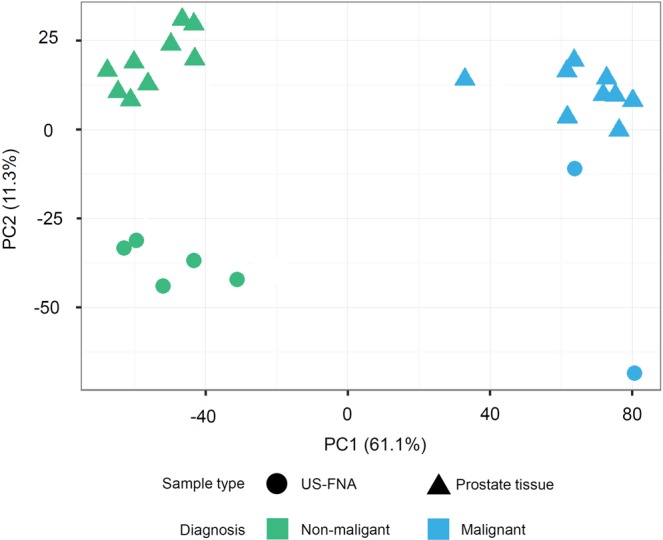
Principal component analysis (PCA-plot) of differentially expressed genes (DEGs). PCA-plot displays summarised data of 3,587 DEGs in 18 prostate tissues (triangle) and seven US-FNA (circle) samples. In general, variances between samples on the x-axis (principal component 1, PC1) separated the data the most (61.1%) and are associated with diagnosis: non-malignant (green) and malignant (blue) samples. Variances on y-axis were lower with 11.3% (principal component 2, PC2) and related to different sampling techniques.

Principal component analysis (PCA) performed on the expression values of the DEGs (see Fig. 5) indicated that most (61.1%) of the variance of the data could be attributed to the diagnosis (non-malignant vs. malignant samples, see PC1 in Fig. 5). Thus, normal to hyperplastic US-FNA and tissue samples were grouped together and separated from malignant US-FNA and tissue samples. In contrast, differences between the sampling procedures contributed only 11.3% to the total variance of the data (see PC2 in Fig. 5).

**Figure 6:**
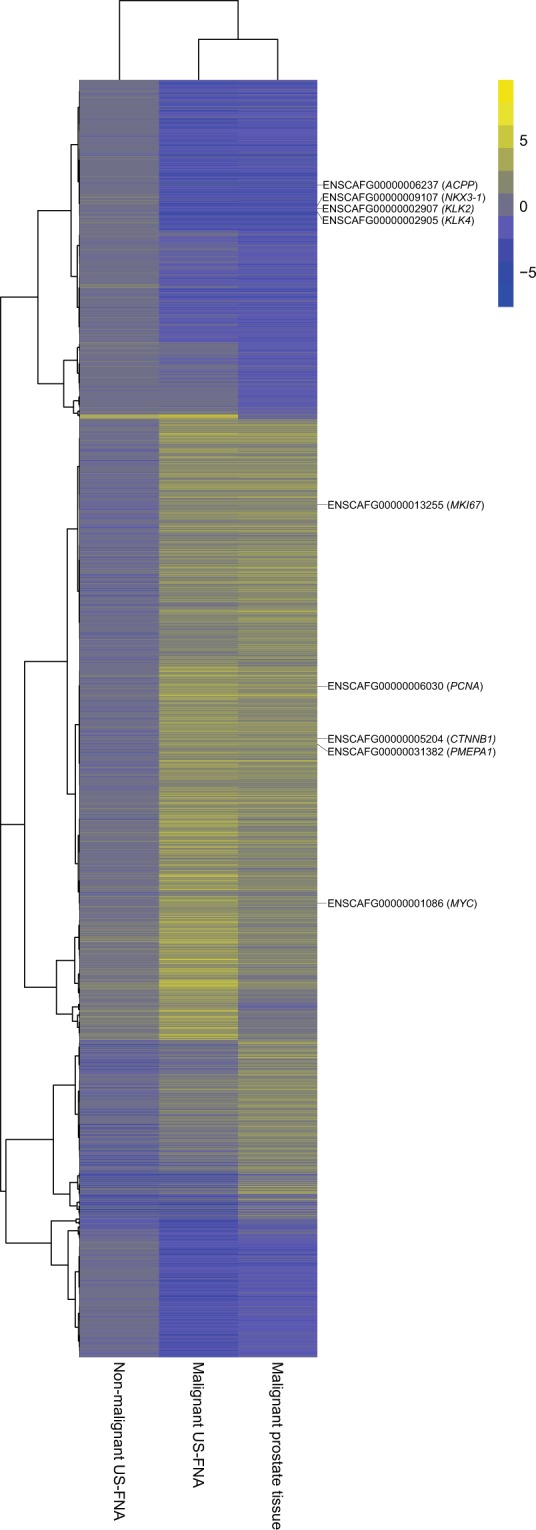
Heatmap of differentially expressed genes (DEGs) in different sample types compared with non-malignant prostate tissue samples. The heatmap and column dendrogram were used to visualise the variances in DEGs across the sample groups based on the average of logarithmic scale base two (log2) fold change. The log2 fold change is displayed on the right side, varying from −11.95 to 11.96. On the right side, Ensembl ID and official gene names show cancer-related genes (e.g., *MYC*, *MKI67*) and genes that were linked to canine prostate diseases (e.g., *NKX3-1*, *KLK4*, *ACPP*) in the heatmap.

Moreover, hierarchical clustering of the fold-changes of the expression values of the DEGs relative to the control group confirmed this result, revealing a largely common molecular profile between malignant US-FNA and malignant tissue samples (see Fig. 6). Specifically, 1,062 genes (30% of the DEGs) were jointly differentially expressed in both malignant US-FNA and malignant tissue samples (see Fig. 4). Most of these genes (612, 58%) were up-regulated. Up-regulated genes included MYC proto-oncogene (*MYC*), catenin beta 1 (*CTNNB1*), prostate transmembrane protein, androgen induced 1 (*PMEPA1*), proliferating cell nuclear antigen (*PCNA*) and marker of proliferation Ki-67 (*MKI67*). The remaining 450 (42%) down-regulated genes included kallikrein-related peptidase 2 (*KLK2*), kallikrein-related peptidase 4 (*KLK4*), NKX3 homeobox 1 (*NKX3-1)* and acid phosphatase prostate (*ACPP*). These have been reported as genes with potential diagnostic value in the literature^33–36^ and their consistent expression in US-FNA and tissue samples supports the use of US-FNA samples for transcriptomic profiling. In addition, only 36 genes (1% of the DEGs) were associated with both non-malignant and malignant aspirates of US-FNAs (see Fig. 4) and not in any way with the diagnoses, and, hence, could be considered as FNA-specific. Of these genes, nine were up-regulated and 27 were down-regulated relative to the control group. Pathway enrichment analysis performed on the 36 DEGs identified significantly overrepresented pathways in up-regulated genes. Four of these genes were involved in pathways associated with blood: Malaria and African trypanosomiasis pathway. Manual annotation (see Materials and Methods) of up-regulated genes identified a total of five globin genes: ENSCAFG00000029904 and ENSCAFG00000032615 as an orthologue of haemoglobin alpha 1 (*HBA1*) and 2 (*HBA2*), ENSCAFG00000029518 and ENSCAFG00000030286 as an orthologue of haemoglobin subunit delta (*HBD*) and haemoglobin beta (*HBB*) and ENSCAFG00000029224 as haemoglobin subunit mu. Therefore, molecular profiling of sampling-associated differences appears to reflect blood contamination in US-FNA samples.

## Discussion

Canine prostate diseases are currently unsatisfactorily characterised at molecular level, hampering early detection strategies^4^. Having the opportunity to further use samples collected for routine diagnostic for molecular profiling is paramount to improve our molecular understanding of diseases^16^. An ideal biopsy procedure should be minimally invasive and allow rapid sampling and comprehensive clinical diagnostics, including molecular and cellular marker analysis^14,27^. In general, the FNA procedure has been shown to meet these requirements^37^. In particular, the present study confirms that FNA samples are a reliable sampling tool for both cytological and molecular diagnosis.

In human oncological medicine, different types of biopsies – including FNA – have been comparatively assessed as samples for NGS applications aimed at optimising the management of patients based on their molecular profiles^38^. In order to address the aggressiveness of human PCa using the Gleason Score, the FNA procedure has been largely replaced in routine diagnostic by multiple core needle biopsies^39^. Nevertheless, aspirates collected by FNA are acknowledged as samples with molecular diagnostic potential^38,40–43^. In dogs, the collection of canine prostate samples is challenging due to the anatomical localisation of the prostate gland^44^. Although histopathological biopsy is described as the gold standard for diagnosis of canine prostate diseases^2,44^ and a histopathological standard terminology is available^45^, the collection of tissue samples has several disadvantages, such as the need for general anaesthesia and the relatively high complication rates^46^. A practicable minimally invasive sampling procedure would be preferable both veterinary research and, especially, in clinical routine^16^. Based on the current state of knowledge, this is the first study to evaluate the feasibility of using samples of the canine prostate obtained *intra vitam* by US-FNA with 22-gauge needles for both cytological diagnosis and RNA-Seq.

In agreement with the literature^2,28^, the US-FNA procedure was well tolerated by 95% of the dogs in the study without the need of general anaesthesia. Complications associated with the US-FNA procedure were transient and mild and in concordance with previous studies^2,47^. The US-FNA procedure proved more challenging in neutered healthy dogs due to the dense and firm prostate tissue structure^48^ caused by androgen deprivation^45,49^ and resulted in generally low quantities of RNA. Nonetheless, the US-FNA procedure yielded adequate quantities of RNA in the case of neutered dogs with PCa, which typically exhibit disease-related changes of the prostate. This is particularly important since neutered dogs have an increased risk to develop canine PCa^50^. These findings indicate that the US-FNA procedure is feasible in diagnostic routine.

In this study, US-FNA samples were split into two subsamples to address conventional and molecular diagnostic questions. An adequate cellularity is crucial for cytology and RNA extraction. The strategy of splitting the aspirates into subsamples generally decreases the cellularity. Varying cellularity can be additionally explained by the non-standardisable conditions during collection and subsampling in the clinical routine. The cellularity of aspirates has been previously analysed in different tumour samples and shown to be affected by the application of suction, the number of needle passes and cell exfoliation^51^. In the present study, 88% of the cytological smears exhibited sufficient cellularity for cytological diagnosis. This was similar to the fraction of the subsamples that met the requirements for RNA-Seq: 93% of US-FNA samples. In concordance with these results, previous studies described the remaining aspirates as a viable source for advanced diagnostic procedures after the preparation of cytological smears^52,53^. In general, the results of this study support the idea of using subsamples to bridge the gap between clinical and molecular diagnosis.

Cytological findings on the sample quality such as necrosis and blood contamination appear to provide important information regarding the viability of molecular analysis. Large amounts of blood are known to influence the quality of cytological smears, hampering an adequate cytological diagnosis^54^. In fact, in a previous study, the researchers opted for not using highly blood contaminated canine FNAs of different tumour entities for RNA extraction^51^. In the present study, 65% of US-FNA samples had moderate to strong blood contaminations, probably related to the aspiration technique^51^ and the use of intravenous needles^55^. Processing and pipetting of contaminated US-FNA samples was challenging and might have led to a decrease in the number of cells. However, US-FNA samples were successfully used for RNA purification by phenol-chloroform extraction. Based on the results in this study, the use of column-based isolation should be avoided without any further lysis steps or reagents. Interestingly, and consistent with the macroscopic findings, five globin genes, which can be associated with erythroid cells^56^, were more highly expressed in US-FNA samples compared with tissue samples. Therefore, blood contamination was detectable at molecular level, thus indicating that RNA-Seq enables *intra vitam* sampling associated effects to be identified. Additionally, there was no evidence that 0.1% of DEGs identified as globin genes in anyway impaired the actual diagnostic for gene expression profiling.

In general, RNA-Seq demands high-quality total RNA^12,15,57^. Of US-FNA samples, 56% met the requirements of RNA-Seq. It has been shown before that RIN values of FNA samples are not always detectable^51^. For precise assessment of RNA quality the Agilent RNA 600 Nano assay requires a minimum RNA concentration of 25 ng/µl^58^. Of the US-FNA samples, 31% (n = 5) selected for quality control were below this threshold, which could explain some of the poor RIN values. Furthermore, the RIN value and the corresponding electropherogram needs to be evaluated. In addition, the nucleases and factors associated with the FNA procedure itself, such as the needle length, can cause RNA degradation^59^. Moreover, this study evaluated the RNA integrity in non-malignant and malignant canine prostate tissue samples with no evidence of a difference. Similar to the results of the present study, one recent study on canine tumour FNA found no significant difference analysing the integrity of RNA from readily exfoliative tumour and non-readily exfoliative tumour FNA. However, non-malignant samples were not part of the above mentioned study^51^.

One previous investigation on human PCa emphasises the importance of biopsy based gene-expression studies and successfully used prostatectomy samples for molecular profiling and additional needle core biopsies for identification of candidate genes^60^. To evaluate the molecular diagnostic potential of canine prostate US-FNA subsamples for further gene expression studies, seven US-FNA samples were subjected to RNA-Seq and their whole transcriptome profiles were compared to those of 18 prostate tissue samples. The main advantage in using NGS is the possibility to comprehensively characterise the transcriptome, allowing rapid profiling of neoplastic diseases for downstream diagnostics of therapeutic intervention^13,16,61^. The findings of the present study indicate that the sampling procedure has a minor impact on the sample’s gene expression profile, since only 36 (1%) of 3,587 DEGs were specifically identified in all US-FNA samples relative to the non-malignant prostate tissue control. Differences on the gene expression level between US-FNA and tissue samples can be explained based on the variability of the individual samples and the diagnosis. In particular, tumour heterogeneity has been described in different cancer types^17,62^, including canine PCa^63^. To minimise these effects and ensure representative material for RNA-Seq analysis as well as adequate diagnosis, the samples were collected under ultrasound guidance and characterised by cytological examination. The molecular profiling of aspirates collected by FNA samples in dogs has been previously performed and compared to matched tissue samples for lymph nodes using microarray technology^31^. Nevertheless, that study differs from the present one in that FNA samples were i) collected only from euthanised dogs (*post mortem*), and ii) not separated into two subsamples for cytological and molecular analysis. Overall, the authors described FNA samples as a reliable source for molecular profiling^31^, which agrees with the results presented here. In this context, US-FNA transcriptomics becomes increasingly attractive as a diagnostic tool. Indeed, malignant and non-malignant US-FNA samples were perfectly separable based on their molecular profiles, matching the cytological diagnosis. In addition, genes that are well known to be deregulated in cancer such as *MYC, MKI67*, *ACPP*, *CTNNB1*, *KLK2*, *KLK4, PCNA*, *PMEPA1*, *NKX3-1*^4,33,64–68^ were also significantly deregulated (and in the same direction) in US-FNA samples, confirming the similarity of US-FNA samples and their prostate tissue counterparts. These genes and other genes with similar molecular profiles are interesting from a diagnostic point of view. In particular, genes like *MYC, MKI67, CTNNB1, PCNA*, *NKX3.1, ACPP* and kallikreins are warranted further investigation as diagnostic markers for cancer and cell proliferation. The combination of US-FNA and RNA-Seq opens up a wide array of possibilities in clinical diagnostics.

In conclusion, the data presented here underline the suitability of the US-FNA procedure as a sampling tool for cytological and molecular diagnostic purposes. To optimise the laboratory workflow, details on cytological examination may be helpful for identifying and processing adequate samples prior to sequencing. This study highlights the strengths of US-FNA as a sampling tool for molecular profiling of the canine PCa. Validation on a larger number of US-FNA samples is warranted to confirm these conclusions. Beyond the use of US-FNA for whole transcriptome analysis, this study provides a solid basis for approaches such as targeted sequencing or qPCR screening of single genes, opening new perspectives for biomarker discovery for early detection and follow-up diagnosis and, eventually, for developing and establishing individualised therapeutic strategies.

## Materials and Methods

### Study design

This study referred to three different sampling types, including US-FNA, PM-FNA and prostate tissue samples (see Fig. 1). Specifically, the term FNA is used for aspiration of a very small amount of tissue or cells. *Intra vitam* aspirated cells, collected by US-FNA were referred to as US-FNA, while aspirates collected by FNA after prostatectomy from euthanised dogs were referred to as PM-FNA training set (see Fig. 1). Aspirates collected by US-FNA were diagnosed cytologically, while those collected by PM-FNA were used to standardise the sampling procedure; both types of aspirates were used for evaluating RNA quantity; a number of aspirates collected by US-FNA were checked on RNA quality and subjected to RNA-Seq (see Fig. 1). The US-FNA sampling procedure was performed prospectively between July 2014 and January 2016 at the Small Animal Clinic of the University of Veterinary Medicine Hannover, Foundation (Germany) in accordance with the German Animal Welfare Guidelines and approved by the Ethics Committee of the State of Lower Saxony, Germany (No. 14/1700).

The term prostate tissue sample is used for structurally and functionally organised cells with a preserved architecture. Prostate tissue samples were collected as matching tissue samples (n = 31) after the PM-FNA procedure from euthanised dogs (see Fig. 1). The sample set was completed by 12 freshly frozen prostate tissue samples from the local tissue bank obtained from between 2002 and 2016. Prostate tissue samples were generally used as control. None of the dogs had been euthanised for the purpose of this study. All dog owners agreed to sample collection.

For a representative set of non-malignant and malignant samples, dogs with symptoms or medical history suggesting a prostate disease were enrolled for clinical evaluation and US-FNA procedure, while euthanised dogs with and without prostate diseases were used for *post mortem* sample collection. Overall, the sample set used in this study comprised 42 US-FNA samples, 31 PM-FNA samples as training set and 43 prostate tissue samples taken from 84 male dogs (see Table 1).

### Standardisation of the sampling procedure using PM-FNA samples as training set

A training set of 31 PM-FNA samples was collected from euthanised dogs during necropsy after prostatectomy (see Fig. 1). Each prostatectomy was performed under sterile conditions within a maximum of two hours after euthanasia. After prostatectomy, *ex vivo* aspiration of prostate cells was performed using a 22-gauge single-use-needle (Terumo, Eschborn, Germany) attached to a 5 mL Luer syringe (Dispomed, Gelnhausen, Germany). The needle was inserted into prostate tissue and moved several times while aspirating. A fraction of the aspirated cells were used to prepare a smear for cytological examination; the remaining cells were expelled to a 1.8 mL Nunc CryoTube (Thermo Fisher Scientific, Nunc-A/S, Roskilde, Denmark). The needle was then rinsed with 50 to 100 µl sterile phosphate-buffered saline (PBS) for collecting the remaining aspirate in the CryoTube. Matched prostate tissue samples were collected from macroscopically representative areas for molecular biological procedures and histopathological classification.

Samples collected for the purpose of molecular biological analysis were immediately snap frozen in liquid nitrogen and stored at −80 °C.

### Histopathology

For histopathological evaluation, prostate tissue samples were fixed in 10% neutral-buffered formalin, embedded in paraffin and examined microscopically on haematoxylin and eosin stained section by a certified pathologist.

### Feasibility of *intra vitam* US-FNA sampling procedure

To evaluate the feasibility of the PM-FNA sampling procedure in clinical routine, a total of 43 dogs were examined *intra vitam* (see Table 1). All dogs underwent general examination. Transabdominal ultrasonography was performed in dorsal recumbency using a Logiq 7 GE Healthcare ultrasound system (General Electric Company, Waukesha, USA). Longitudinal and transverse images of the prostate, urinary bladder, sub-lumbar lymph nodes and testes were evaluated. The US-FNA procedure of the prostate was performed using the previously described standard protocol for US-FNA of the canine prostate^69^. Differences of parenchyma echogenicity^3,30^ were used for identification and collection of representative samples. The US-FNA samples were processed as the above mentioned the PM-FNA sampling protocol. The US-FNA procedure was repeated up to three times in cases where samples were suspicious of having inadequate material. Processing of aspirated samples and long-term storage were performed in accordance with the PM-FNA procedure.

Two dogs were euthanised due to an advanced stage of prostate carcinoma. In one of these dogs, the US-FNA procedure was repeated immediately after euthanasia (see Table 2). For both dogs, additional PM-FNA and matched prostate tissue samples were accessible during necropsy.

### Cytology

Cytological smears were air dried and stained in accordance with Pappenheim standard operational procedures and examined within one hour by an experienced cytologist. Previous reports on prostate cytology and general cytological categories^28,70,71^ were used to classify e.g. cluster of uniform cells as normal to hyperplastic, inflammatory cells as indicator for inflammation and samples with cells presenting criteria of malignancy as malignant. Cytological smears with fewer than ten cells in low power field using a 100-fold magnification were classified as non-diagnostic.

### Isolation and quantification of total RNA from PM-FNA and US-FNA samples

Total RNA from the PM-FNA training set (n = 31) was isolated in accordance with the manufacturer’s protocol of Qiagen AllPrep™DNA/RNA Micro Kit (Qiagen, Hilden, Germany). For lysis and homogenisation, Buffer RLT Plus and QIAshredder spin column (Qiagen, Hilden, Germany) were used and purified RNA was eluted RNAse-free water.

For *intra vitam* collected US-FNA samples (n = 42), the method of RNA isolation was adjusted to phenol-chloroform extraction (n = 40), since column-based isolation of total RNA from two US-FNA samples failed due to clotted blood. A semi-quantitative scoring was used prior to RNA isolation to assess blood contamination in 40 US-FNA samples macroscopically: Score 0 was used for samples without macroscopically visible contaminations of blood; score 1 comprised samples with almost invisible contamination of blood; score 2 included light red coloured samples for weak but clearly visible blood contamination; score 3 included red coloured samples, describing a moderate contamination and score 4 deep red samples indicating strong blood contamination.

Phenol-chloroform extraction of RNA from remaining aspirates collected by US-FNA (n = 40) was performed using QIAzol Lysis Reagent with Qiagen miRNeasy Micro Kit (Qiagen, Hilden, Germany) eluted RNase-free water. RNA quantity of US-FNA and PM-FNA samples was determined using the Qubit® RNA HS Assay Kit (ThermoFisher Scientific, Waltham, USA) with Qubit 2.0 Fluorometer.

### RNA extraction from prostate tissue samples

For isolating total RNA from 10-20 mg prostate tissue samples (n = 43), AllPrep® DNA/RNA/miRNA Universal Kit (Qiagen, Hilden, Germany) was used in accordance with the manufacturer’s protocol. Tissue samples were disrupted utilising Buffer RLT Plus and 5 mm stainless steel beads by TissyeLyser II (Qiagen, Hilden, Germany). Total RNA was eluted in 30 µl RNase free-water and RNA quantity was measured on a Take3^TM^ Multi-Volume Plate by Synergy™II multi-mode reader, evaluated by Gen5™ Microplate Data Analysis Software (Biotek, Bad Friedrichshall, Germany).

### Sample selection and evaluation of RNA quality

US-FNA and prostate tissue samples for RNA-Seq were selected in accordance with multiple criteria. First, they were required to contain a minimum of 10 ng and a maximum of 1 µg of total RNA. To ensure their comparability, the samples were further selected based on their histopathological and cytological diagnosis (see Fig. 1) and non-malignant and malignant US-FNA aspirates and prostate tissue samples were the subject of focus. The non-malignant sample group comprised normal to hyperplastic samples and included 12 US-FNA and nine prostate tissue samples. The malignant group included four US-FNA and nine prostate tissue samples (see Table 2). The PM-FNA training set was excluded.

The RNA quality was measured using the RNA 6000 Nano LabChip on an Agilent Bioanalyzer 2100 (Agilent Technologies, Santa Clara, USA), based on the 18S to 28S ribosomal ratio provided as electropherogram. The RIN value was determined with the Agilent 2100 Expert software and ranged from 1 (most degraded RNA) to 10 (most intact RNA). For samples with low RIN values, details in the electropherogram were used for further evaluation of RNA degradation.

### Statistical analysis of RNA quantity and quality

Statistical analysis of RNA quantity and quality was performed using SAS Enterprise Guide 7.1 (SAS Institute Inc., Cary, North Carolina, USA). The Shapiro-Wilk test was applied to confirm the normal distribution of the data. The influence of neutering and diagnosis on RNA quantity in prostate tissue samples was evaluated with the two-sample t-test. Wilcoxon’s signed-rank test was used to compare the differences between US-FNA aspirates and the PM-FNA training set. A two-sample t-test was performed to analyse differences of RIN-values between non-malignant and malignant tissue samples. Significance level was defined as p < 0.05.

### RNA-sequencing, mapping and differential expression analysis

Samples with a RIN value ≥ 5.5 (US-FNA n = 7, prostate tissue n = 18) were then subjected to NGS library preparation (see Table 2) using the NEBNext Ultra RNA preparation kit (New England Biolabs, Ipswich, USA). Single-read sequencing was conducted on an Illumina NextSeq500 with a read length of 75 bp (Illumina, San Diego, USA). Sequencing data have been deposited in the Gene Expression Omnibus database (accession identifier GSE122916)^72^.

Raw sequencing reads were quality checked using FastQC^73^. Adaptors and low-quality bases were trimmed using Trimmomatic (v0.32^74^) with the following parameters: LEADING:3 TRAILING:3 SLIDINGWINDOW:4:15 MINLEN:36. Trimmed reads were then mapped to the CanFam3.1 assembly of the dog genome using TopHat2^75^ with default parameters and supplying the Ensembl GTF annotation (Ensembl release 85^76^) through the -G option. This resulted in an average of ~14 million pairs of mapped reads per sample.

Read counts for each protein-coding gene were calculated for each sample using the htseq-count tool of the Python package HTSeq^77^ with default parameters. The sample groups: non-malignant US-FNA, malignant prostate tissue and malignant US-FNA were each independently tested against non-malignant prostate tissue (control) for differential expression with the R/Bioconductor package DESeq2^78^. *p*-values were adjusted for multiple testing using the false discovery rate (FDR). Genes with an FDR < 0.00001 in any of the three comparisons were considered as DEGs.

### Manual annotation of single genes

Nucleotide sequences of Ensembl gene IDs^76^ not associated with any Official Gene Symbol were aligned for identifying sequence homologies based on Ensembl IDs using the online BLAST (Basic Local Alignment Search Tool) interface provided by NCBI (https://blast.ncbi.nlm.nih.gov/Blast.cgi ^79,80^).

### Principal component analysis and hierarchical Clustering

DEGs were used to perform a PCA to summarise and visualise variances across all samples. Specifically, PCA was performed on library size-normalised regularised log-transformed expression values, which were computed using the rlogTransformation() function in the R/Bioconductor DESeq2 package.

Fold-changes relative to non-malignant prostate tissue of DEGs were hierarchically clustered using complete linkage and Euclidean distance (genes) or a Pearson correlation-based distance measure (samples). On basis of fold-changes, a list of well-studied genes was used for further profiling of US-FNA in comparison with prostate tissue samples.

### Pathway analysis

DEGs identified exclusively in US-FNA samples were analysed on biological pathway. This pathway analysis was performed with DAVID (Database for Annotation, Visualization and Integrated Discovery Functional Annotation Tool^81,82^), using the 19,856 Ensembl IDs corresponding to all protein-coding genes annotated in the dog genome (Ensembl release 85^76^) as background and a FDR ≤ 5% cut-off for significance.
